# Supplementary material for: mRNA vaccines encoding influenza virus hemagglutinin (HA) elicits immunity in mice from influenza A virus challenge
Source: PLoS One. 2024 Apr 18;19(4):e0297833. doi: 10.1371/journal.pone.0297833 (PMC11025922; doi:10.1371/journal.pone.0297833)
Supplement: Attachment — Submitted filename: Response to Reviewers.docx [file pone.0297833.s001.docx]

December 7, 2023

Dear Editor: We are submitting the revised Manuscript Ref. No.:  PONE-D-23-35405

mRNA Vaccines Encoding Influenza Virus Hemagglutinin (HA) Elicits Immunity in Mice from Influenza A Virus Challenge. We have responded to the journal comments and the referee comments and revised the manuscript for resubmission. We look forward to receiving your comments.

Responses to Journal Comments:

1. Please ensure that your manuscript meets PLOS ONE's style requirements, including those for file naming. The PLOS ONE style templates can be found at 
<https://journals.plos.org/plosone/s/file?id=wjVg/PLOSOne_formatting_sample_main_body.pdf> and 
<https://journals.plos.org/plosone/s/file?id=ba62/PLOSOne_formatting_sample_title_authors_affiliations.pdf>

We thank the journal for their comment. We have addressed the formatting issues.

2. Did you know that depositing data in a repository is associated with up to a 25% citation advantage (<https://doi.org/10.1371/journal.pone.0230416>)? If you’ve not already done so, consider depositing your raw data in a repository to ensure your work is read, appreciated and cited by the largest possible audience. You’ll also earn an Accessible Data icon on your published paper if you deposit your data in any participating repository (<https://plos.org/open-science/open-data/#accessible-data>).

We thank the journal for their comment. I have uploaded the data to ‘Open Science Framework’ DOI 10.17605/OSF.IO/S6Z7X

3. To comply with PLOS ONE submissions requirements, in your Methods section, please provide additional information regarding the experiments involving animals and ensure you have included details on (1) methods of sacrifice,  and (2) efforts to alleviate suffering.

We thank the journal for their comment. We have added information on scoring of clinical symptoms and the method of euthanasia in lines 330-333. If more additional information is needed, we are happy to add more details about any specific procedures that the journal requests.

4. Thank you for stating the following financial disclosure: "Immorna-funded the research study."

Please state what role the funders took in the study.  If the funders had no role, please state: "The funders had no role in study design, data collection and analysis, decision to publish, or preparation of the manuscript."

If this statement is not correct you must amend it as needed.

Please include this amended Role of Funder statement in your cover letter; we will change the online submission form on your behalf.

We thank the journal for their comment. We have added the statement in our cover letter.

5. Thank you for stating the following in the Competing Interests section: "The authors Thornhill-Wadolowski, Feng, Bugno, and Truax are employees of Immorna who provided the mRNA-LNP vaccines."

We note that one or more of the authors are employed by a commercial company: Immorna

     1. Please provide an amended Funding Statement declaring this commercial affiliation, as well as a statement regarding the Role of Funders in your study. If the funding organization did not play a role in the study design, data collection and analysis, decision to publish, or preparation of the manuscript and only provided financial support in the form of authors' salaries and/or research materials, please review your statements relating to the author contributions, and ensure you have specifically and accurately indicated the role(s) that these authors had in your study. You can update author roles in the Author Contributions section of the online submission form.

Please also include the following statement within your amended Funding Statement.

“The funder provided support in the form of salaries for authors [insert relevant initials], but did not have any additional role in the study design, data collection and analysis, decision to publish, or preparation of the manuscript. The specific roles of these authors are articulated in the ‘author contributions’ section.”

If your commercial affiliation did play a role in your study, please state and explain this role within your updated Funding Statement.

We thank the journal for their comment. We have added the statement in our cover letter.

     2. Please also provide an updated Competing Interests Statement declaring this commercial affiliation along with any other relevant declarations relating to employment, consultancy, patents, products in development, or marketed products, etc.

Within your Competing Interests Statement, please confirm that this commercial affiliation does not alter your adherence to all PLOS ONE policies on sharing data and materials by including the following statement: "This does not alter our adherence to  PLOS ONE policies on sharing data and materials.” (as detailed online in our guide for authors <http://journals.plos.org/plosone/s/competing-interests>). If this adherence statement is not accurate and there are restrictions on sharing of data and/or materials, please state these. Please note that we cannot proceed with consideration of your article until this information has been declared.

Please include both an updated Funding Statement and Competing Interests Statement in your cover letter. We will change the online submission form on your behalf.

We thank the journal for their comment. We have added the statement in our cover letter.

6. We note that you have indicated that data from this study are available upon request. PLOS only allows data to be available upon request if there are legal or ethical restrictions on sharing data publicly. For more information on unacceptable data access restrictions, please see <http://journals.plos.org/plosone/s/data-availability#loc-unacceptable-data-access-restrictions>.

In your revised cover letter, please address the following prompts:

a) If there are ethical or legal restrictions on sharing a de-identified data set, please explain them in detail (e.g., data contain potentially sensitive information, data are owned by a third-party organization, etc.) and who has imposed them (e.g., an ethics committee). Please also provide contact information for a data access committee, ethics committee, or other institutional body to which data requests may be sent.

b) If there are no restrictions, please upload the minimal anonymized data set necessary to replicate your study findings as either Supporting Information files or to a stable, public repository and provide us with the relevant URLs, DOIs, or accession numbers. For a list of acceptable repositories, please see <http://journals.plos.org/plosone/s/data-availability#loc-recommended-repositories>.

We will update your Data Availability statement on your behalf to reflect the information you provide.

We thank the journal for their comment. I have uploaded the data to ‘Open Science Framework’ DOI 10.17605/OSF.IO/S6Z7X

7. PLOS requires an ORCID iD for the corresponding author in Editorial Manager on papers submitted after December 6th, 2016. Please ensure that you have an ORCID iD and that it is validated in Editorial Manager. To do this, go to ‘Update my Information’ (in the upper left-hand corner of the main menu), and click on the Fetch/Validate link next to the ORCID field. This will take you to the ORCID site and allow you to create a new iD or authenticate a pre-existing iD in Editorial Manager. Please see the following video for instructions on linking an ORCID iD to your Editorial Manager account: <https://www.youtube.com/watch?v=_xcclfuvtxQ>

We thank the journal for their comment. There was an old account connected to my ORCID ID. I have corrected the issue and linked my account.

8. We note that you have included the phrase “data not shown” in your manuscript. Unfortunately, this does not meet our data sharing requirements. PLOS does not permit references to inaccessible data. We require that authors provide all relevant data within the paper, Supporting Information files, or in an acceptable, public repository. Please add a citation to support this phrase or upload the data that corresponds with these findings to a stable repository (such as Figshare or Dryad) and provide and URLs, DOIs, or accession numbers that may be used to access these data. Or, if the data are not a core part of the research being presented in your study, we ask that you remove the phrase that refers to these data.

We thank the journal for their comment. This section of text has been removed from the manuscript due to reviewer comments.

Response to Reviewer Comments:

Reviewer 1:

Major comments:
1. The introduction needs expanding on other studies that have used mRNA vaccines for influenza to put the work into context. At present it is one paragraph of introduction and then another paragraph summarising the current studies.

We thank the reviewer for their comment. We have added a paragraph discussing other mRNA influenza vaccine studies.

2. The work could be more ambitious in scope – for example challenge with a different virus to show the benefits of combining the mRNA vaccines. The cross neutralization data is of interest, but the rest of the manuscript is light on experiments – it could be compressed to 2 or maybe 3 figures at the moment.

We thank the reviewer for their comment. We plan to expand our experiments with future studies, but we feel that the number of figures and tables is appropriate for this manuscript. We will take this comment into consideration to improve the studies for our future manuscripts.

Minor comments:
1. Rather than saying group 1-10 in numbers put what they received on the graphs, it is very hard to read.

We thank the reviewer for their comment. We have added labels for clarification.

2. Why are only some groups challenged with virus

We thank the reviewer for their comment. We had excluded Groups 4 and 5 from several figures due to mice in each group having to be sacrificed. We have included the weight loss data for the mice in for these groups in figure 1 and added clarification in lines 113-15 explaining these group exclusions from figure 2.

3. Y axis scale is wrong on the weight loss graph.

We thank the reviewer for their comment. We have corrected this error.

4. Figures 3 and 4 put the virus strains above each graph

We thank the reviewer for their comment. We have added the stains for clarification.

5. Figure 5 present as individual points

We thank the reviewer for their comment. Due to several comments about this figure, we have completely removed the figure from the manuscript.

Reviewer 2:

1.Materials and methods, How many animals were used in this experiment? How many animals were used in each group and do they all have pathogenic symptoms?

We thank the reviewer for their comment. We have added information on lines 289-299.

2.Why did the author use A/California/04/2009 as the challenge strain? Is this strain still representative at present? What would happen if the current epidemic strain were used for the challenge?

A/California/04/2009 is a well-established challenge virus for influenza vaccine studies (doi: 10.1016/j.antiviral.2011.02.001, doi: 10.1128/JVI.00076-13). A/California/04/2009 is a pH1N1 virus that is the same lineage that still circulates today. In our study, we wanted to challenge with an antigenically distinct H1N1 virus to ensure that our vaccine was electing a broadly cross-reactive antibody response and not a narrow antibody response only to the HA antigen that was used in the vaccine.

3.Table 2, A/Dar/21 and B/Phu/13 strains produced low antibody titers, especially group 8, Why?

We thank the reviewer for their comment. H3N2 and Influenza B strains are much less immunogenic than H1N1 strains. Group 8 is a single vaccination while all other groups received 2 vaccinations which is likely the reason for the lower titers.

4.Figure 1, Why the weight value of group 3 was low, even lower than the control (group 1)?

We thank the reviewer for their comment. While it appears that there is slight difference on days 4 and 5 between these groups (as well as groups 4 and 5 that were added to this figure), these differences are not statistically significant.

5.Figure 2, why the vaccine provided complete protection in groups 2 and 6? Is there any correlation between immune and challenge strains?

We thank the reviewer for their comment. Group 2 and 6 had high HAI titers to Cal/09 (Figure 4). HAI titers are often highly correlated with low lung viral titers. Groups 2 and 6 likely had Ab titers high enough to limit viral replication to below the limit of detection for the plaque assay.

6.Figure 3 D and Figure 4 D, it was low levels of HAI antibodies immunized with the Phuket/3073/2013 strain in group 5, why?

We thank the reviewer for their comment. There is a single amino acid mismatch between the vaccination strain and the Phuket/3073/2013 virus used in figures 3 and 4. This is stated in lines 243-245.

7.Figure 4 D and H, The mixed vaccine also did not produce significant antibodies. Why? It is necessary to discuss the characteristics of the strain.

We thank the reviewer for their comment. The amino acid mutation is likely causing lower titers in panel D. The results in panel H are showing an antigenically distinct B/Yamagata strain. These results are similar to the antigenically distinct B/Victoria strain in panel G. None of the mixed vaccine produced high antibody titers to any of the antigenically distinct IBVs. This is likely due to the low immunogenicity of IBV HA proteins.

8.Figure 5, where are 4 and 5 groups?

We thank the reviewer for their comment. Due to several comments about this figure, we have completely removed the figure from the manuscript.

9.Figure 5, I think that it may not provide significant result. The number of T cells would significant change only in consumptive or critical diseases. It is more meaningful to analyze the number of T cells that can secrete specific cytokines.

We thank the reviewer for their comment. Due to several comments about this figure, we have completely removed the figure from the manuscript.
